# HPV 16E7 and 48E7 proteins use different mechanisms to target p130 to overcome cell cycle block

**DOI:** 10.1186/s12985-015-0460-8

**Published:** 2016-01-04

**Authors:** Nurshamimi Nor Rashid, Zi Ling Yong, Rohana Yusof, Roger J. Watson

**Affiliations:** Section of Virology, Department of Medicine, Imperial College London, London, UK; Department of Molecular Medicine, Faculty of Medicine, University of Malaya, 50603 Kuala Lumpur, Malaysia; Institute of Biomedical Research, College of Medical and Dental Sciences, University of Birmingham, Birmingham, UK

**Keywords:** p130, Retinoblastoma like protein 2 (RBL2), DREAM complex, Human papillomavirus E7 oncoprotein, Cervical cancer

## Abstract

**Background:**

Retinoblastoma like protein 2 (RBL2) or p130 is a member of the pocket protein family, which is infrequently mutated in human tumours. Its expression is posttranscriptionally regulated and largely G0 restricted. We have previously shown that E6/E7 oncoproteins encoded by human papillomavirus (HPV) type 16, which is a high-risk type for cervical cancer development, must target p130 to promote the host cell to exit from quiescence (G0) state and enter S phase of the cell cycle. P130 is associated with the DREAM (DP, RB-like, E2F and MuvB) complex in G0/G1, which prevents S phase progression by repressing transcription of E2F-regulated genes. E7 proteins could potentially disrupt the p130-DREAM complex through two known mechanisms: direct interaction with p130 or induction of cyclin dependent kinase 2 (CDK2) phosphorylation by interacting with its inhibitor, p21^CIP1^.

**Methods:**

In this study we have used p130 mutants deficient in binding the E7 LXCXE domain (p130mE7), unphosphorylatable by CDK2 (p130PM22) or a combination of both (p130PM22/mE7) to investigate these mechanisms used by E7 proteins to disrupt the p130-DREAM complex and promote cell cycle progression.

**Results:**

We found that HPV16 E7 binding to p130 through its LXCXE domain was absolutely required to disrupt p130-DREAM to promote S phase of the cell cycle, as HPV16 E7 was unable to suppress p130mE7 but could suppress p130PM22. In contrast, the E7 protein encoded by a cutaneous HPV type that lacks a functional LXCXE domain, HPV 48 E7, was also able to disrupt p130-DREAM to promote cell cycling, but through the alternative mechanism. Thus, HPV48 E7 could suppress a cell cycle block imposed by p130mE7, but was unable to suppress p130PM22.

**Conclusions:**

Overall, these results indicate that suppression of p130 is required for HPV-induced cell cycling, and that different HPV E7 proteins can use alternative mechanisms to achieve this.

**Electronic supplementary material:**

The online version of this article (doi:10.1186/s12985-015-0460-8) contains supplementary material, which is available to authorized users.

## Background

In women, cervical cancer is the second most frequent cancer worldwide and the leading cause of cancer related deaths in developing countries [1]. As an example, cervical cancer ranks as the second most frequent cancer among women in Malaysia, in women between 15 and 44 years of age. Data are not yet available on the HPV burden in the general population of Malaysia [2]. HPV are associated with over 99 % of all cervical cancer, 40-50 % of penile and vulvar cancers and greater than 20 % of head and neck cancers [3]. HPV types which are associated with these mucocutaneous sites are classified as high risk (HR) or low risk (LR) depending on their ability to cause cancer. HPV16, 18, 31, 33 and 45 are HR types commonly associated with malignancies [4], whereas HPV6, 11, 42 and 43 are classified as LR and can cause condyloma acuminata (genital warts) [5]. Many other HPV types are associated only with cutaneous epithelia, and these include HPV4, 48, 50, 65 and 88 which are found predominantly in sites extensively exposed to sun, in both lesions and healthy skin samples [6]. There are also several other cutaneous types of HPVs (1, 2, 3, 4, 10, 27, 28, 57) which are the major causes of skin warts.

The E6 and E7 oncoproteins encoded by HR HPV types are responsible to immortalize human keratinocytes through inactivation of p53 and pRB tumour suppressor proteins, respectively [7]. The E6 and E7 proteins are consistently expressed in cancer cells and inhibiting their expression blocks the malignant phenotype [8, 9]. They are independently able to immortalize various human cell types in tissue culture, but efficiency is increased when they are expressed together [10, 11]. However, E6 and E7 from low risk viruses lack immortalization activity, providing a direct correlation between their *in vitro* properties and the potential carcinogenicity of the HPV type. HR HPV E7 proteins contain a LXCXE motif and a C-terminal domain which synergistically form a high affinity bivalent interaction that both anchors the E7 protein to pRB and dissociates the associated E2F transcription factor [12]. HPV16 E7 preferentially binds to p130 in serum-starved quiescent human fibroblasts [13]. It is notable that the cutaneous HPV10, 48 and 60 types encode E7 proteins which have incomplete LXCXE motifs and a low affinity for the pocket proteins; however, all have some ability to stimulate proliferation and progression into S phase [14], suggesting that direct interaction with pocket proteins is not required for this property.

P130 and its related proteins, pRB and p107 are important regulators of cell cycle progression, senescence, development and differentiation. These proteins share a distinct pocket domain necessary for binding E2F transcription factors and LXCXE motif-containing cellular proteins, including the D-type cyclins and histone deacetylases (HDACs). In their hypo- or unphosphorylated forms, the pocket proteins negatively regulate cell cycle progression through interaction with E2F/DP heterodimers and the recruitment of HDACs that promote chromatin condensation and repress transcription [15].

A multiprotein unit has been identified in human complexes, termed DREAM whose composition is regulated at distinct phases of the cell cycle [16]. The core DREAM complex contains Lin9, Lin37, Lin54, Lin52 and RbAp48 (the human homologues of *Drosophila* Mip130, Mip40, Mip120, dLin52 and Caf1p55, respectively). In G0/G1, DREAM binds to E2F4 and either p130 or p107 to repress transcription of E2F target genes regulating the G1/S transition. In S/G2, DREAM switches to B-myb to activate genes required for G2/M transition and mitosis.

**Fig. 1:**
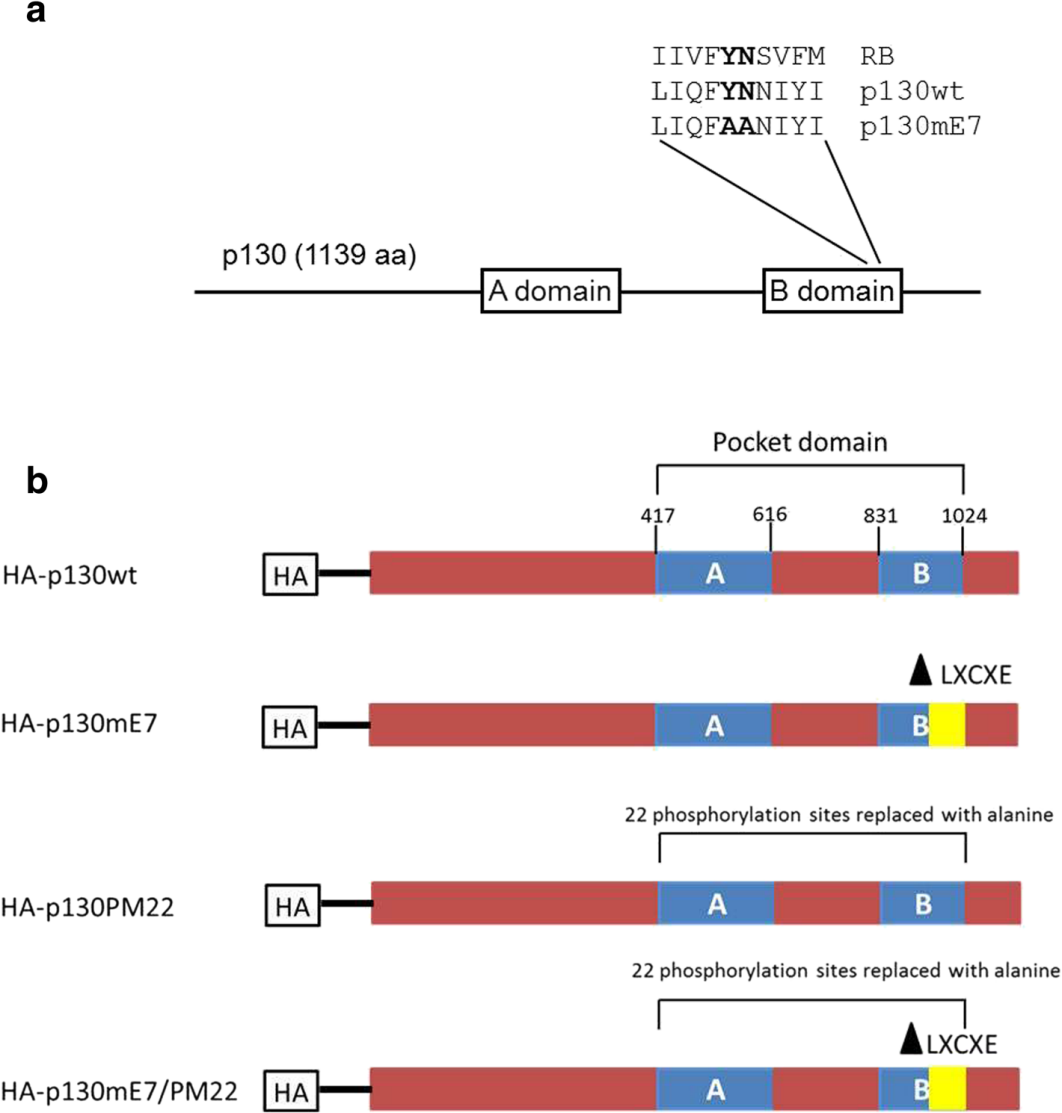
Schematic summary of the constructed p130 mutants based on the p130wt. **a** Schematic of the 1139 amino acid human p130 protein, showing the A and B domains of the pocket. Also indicated is the region of the B domain conserved between RB and p130 which was mutated in p130mE7. In p130mE7, amino acids Y1009 and N1010 were changed to alanines. **b** p130wt has two pocket domains which are A and B for viral oncoproteins binding and cyclin dependent kinase phosphorylation. The p130mE7 mutant was designed based on the work of Dick and Dyson (2002), who showed that a surface of the pRB B pocket was critical for binding proteins containing the L-X-C-X-E motif. This region of the pRB B pocket is partially conserved in p130, and two critical conserved amino acids (leucine and cysteine) in p130 were replaced with alanine by *in vitro* mutagenesis. The p130PM22 mutant has been mutated at the 22 CDK phosphorylation sites by replacing the phosphorylated serine and threonine with alanine (Farkas et al., 2002). The p130mE7/PM22 protein was mutated at both the HPV E7 binding and phosphorylation sites. All p130 mutants and a control wild-type p130 were tagged with HA to differentiate between endogenous and ectopically expressed p130

In this study, we investigated how representative E7 proteins from HR mucocutaneous and cutaneous HPV types target p130. We constructed a p130 mutant that is defective in binding the HPV E7 LXCXE motif (p130mE7) based on conserved amino acids shown previously to be critical for pRB binding to LXCXE-containing viral oncoproteins [17] and took advantage of another mutant which cannot be phosphorylated by CDK (p130PM22) [18] (Fig. 1). In addition, a double mutant (p130mE7/PM22) was constructed. The p130 mutants and a control wild-type p130 were co-transfected with HPV16 and 48 E7 into cell lines. Our results suggest that HPV E7 proteins can use alternative mechanisms to overcome p130-DREAM function in order to promote cell cycling.

## Results and discussion

To construct a p130 mutant unable to bind the E7 LXCXE motif (p130mE7) we replaced two amino acids in the B pocket that are conserved in pRB and shown previously to be essential for LXCXE-binding [17]. We first compared the properties of p130mE7 the unphosphorylatable p130PM22 mutant and the p130mE7/PM22 double mutant with wild type p130 (p130wt) in CaSki and C33a cervical carcinoma cell lines: CaSki cells express HPV16 E7 at high levels, whereas C33a cells are HPV-negative. The transfected p130 proteins were distinguished from endogenous p130 by virtue of an HA tag. Immunoblotting with the p130 antibody did not show increased expression of p130 in the transfected cells, suggesting that the HA-tagged wild-type p130 and the mutants were expressed at a relatively low level, compared to endogenous p130 (Additional file 1: Figure S1a). We found that all p130 mutants and p130wt were equally expressed in the HPV-negative C33a cells, as none of the p130 proteins could be targeted by E7 in this cell line (Additional file 1: Figure S1b). In contrast, neither p130 wt nor p130PM22 were detected in CaSki cells, whereas the p130mE7 and p130PM22/mE7 mutants were both detectable on the HA antibody blot (Additional file 1: Figure S1b). This finding strongly suggested that the p130mE7 mutation prevented binding to 16E7 in Caski cells and thus protected it from degradation. Our *in vitro* binding data confirmed that 16E7 binds to wild type p130, but not to p130mE7 (Additional file 2: Figure S2a). On the other hand, CDK phosphorylation of p130 did not appear to be a major factor in degradation of this protein in Caski cells, as the p130PM22 mutant was not protected.

**Fig. 2:**
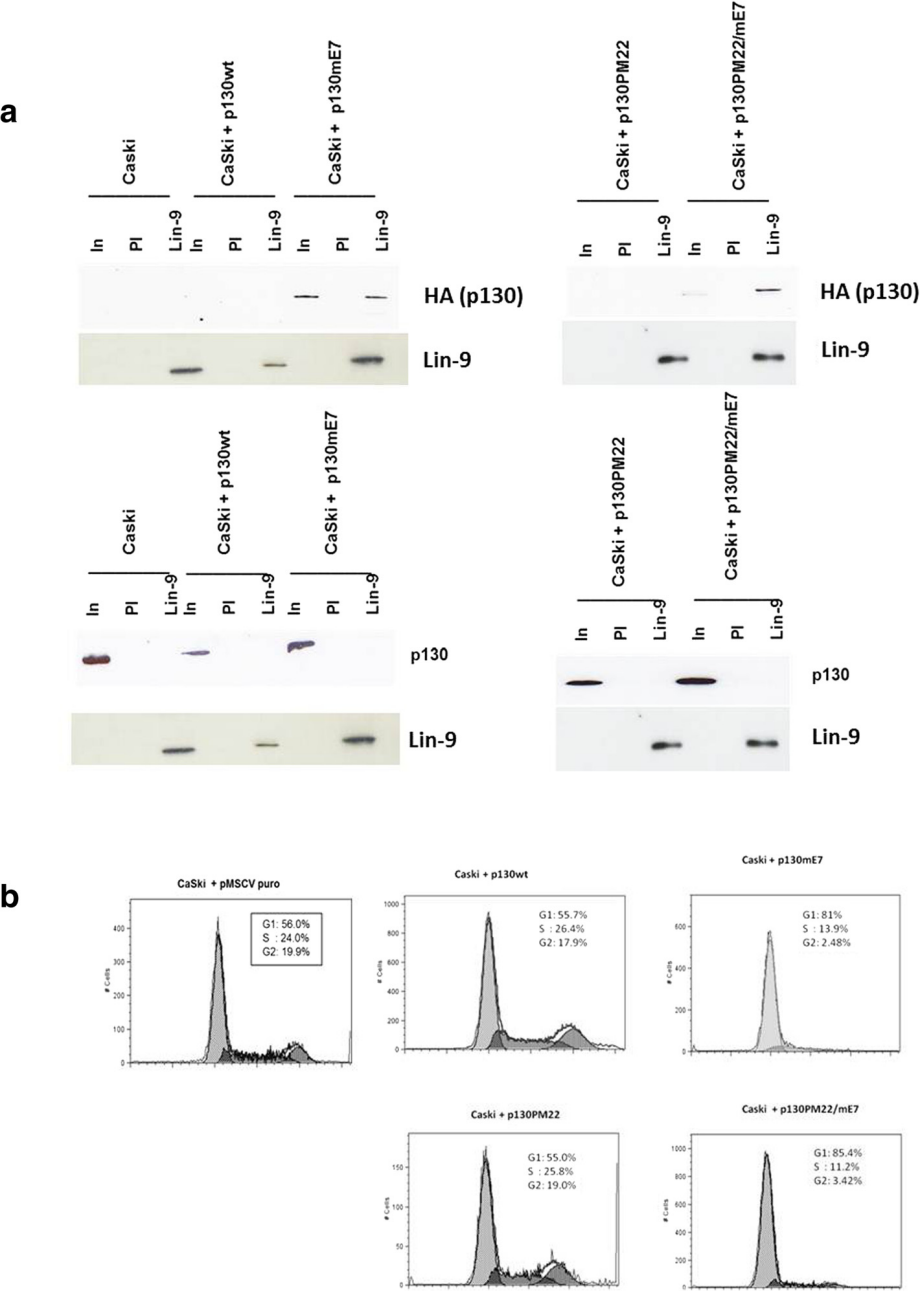
Formation of the p130-DREAM complexes in CaSki expressing p130mE7 and p130PM22/mE7. **a** Nuclear lysates from the untrasfected CaSki cells (CaSki) or CaSki cells transfected with pMSCV puro constructed with p130 wt, p130 mE7, p130PM22 and p130mE7/PM22 were immunoprecipitated with pre-immune serum (PI) or Lin-9 antibodies and were blotted using p130 (Santa Cruz) and HA (Roche) antibodies. The input (In) control comprised of the lysates used for immunoprecipitation. The image is representative of four independent experiments. **b** Flow cytometry of propidium iodide stained CaSki cells transfected with pMSCV puro constructed with p130 wt, p130mE7, p130PM22 and p130mE7/PM22 and pMSCV puro vector only as a control. The estimated percentages of cells in G1, S and G2/M phase are shown

To investigate whether or not the p130mE7 and p130PM22/mE7 mutants could complex with DREAM in CaSki cells, the nuclear lysates from transfected cells were co-precipitated with Lin-9 antibody and probed on western blots using HA and p130 antibodies. As anticipated from the differential expression of p130 proteins (Fig. 2), the results showed that p130mE7 and p130PM22/mE7 were formed into p130-DREAM complexes (Fig. 2a), whereas no p130-DREAM complexes were detected on this blot with p130wt or the p130PM22 mutant. These results further showed that mutation of the B pocket in p130mE7 and p130PM22/mE7 did not affect interactions with DREAM. Unexpectedly, none of the HA-tagged p130 proteins could be detected in DREAM complexes using the p130 antibody on western blots (Fig. 2a), again suggesting they were expressed at relatively low levels.

To test whether transfection with p130wt or any of the mutants had effects on the cell cycle in CaSki cells, transfected cells were harvested, stained with propidium iodide and analysed by flow cytometry. The p130mE7 and p130PM22/mE7 mutants were both able to arrest CaSki cells at G0/G1, with 81 % and 85.4 % at this stage, respectively, compared to 56 % G0/G1 cells in control cells transfected with the empty vector. In contrast, neither p130wt nor p130PM22 had any effect on the cell cycle, with 55.7 % and 55 % at G0/G1, respectively. These results reflected the ability of p130mE7 and p130PM22/mE7 to form DREAM complexes (Fig. 2b), and demonstrated that the levels of complex formation achieved with these mutants was sufficient to cause cell cycle arrest. It is notable that p130mE7 was as efficient as the p130PM22/mE7 double mutant in this cell cycle arrest assay, suggesting that direct interaction of 16E7 with p130 through its LXCXE motif is the key determinant in promoting the cell cycle in CaSki cells.

**Fig. 3:**
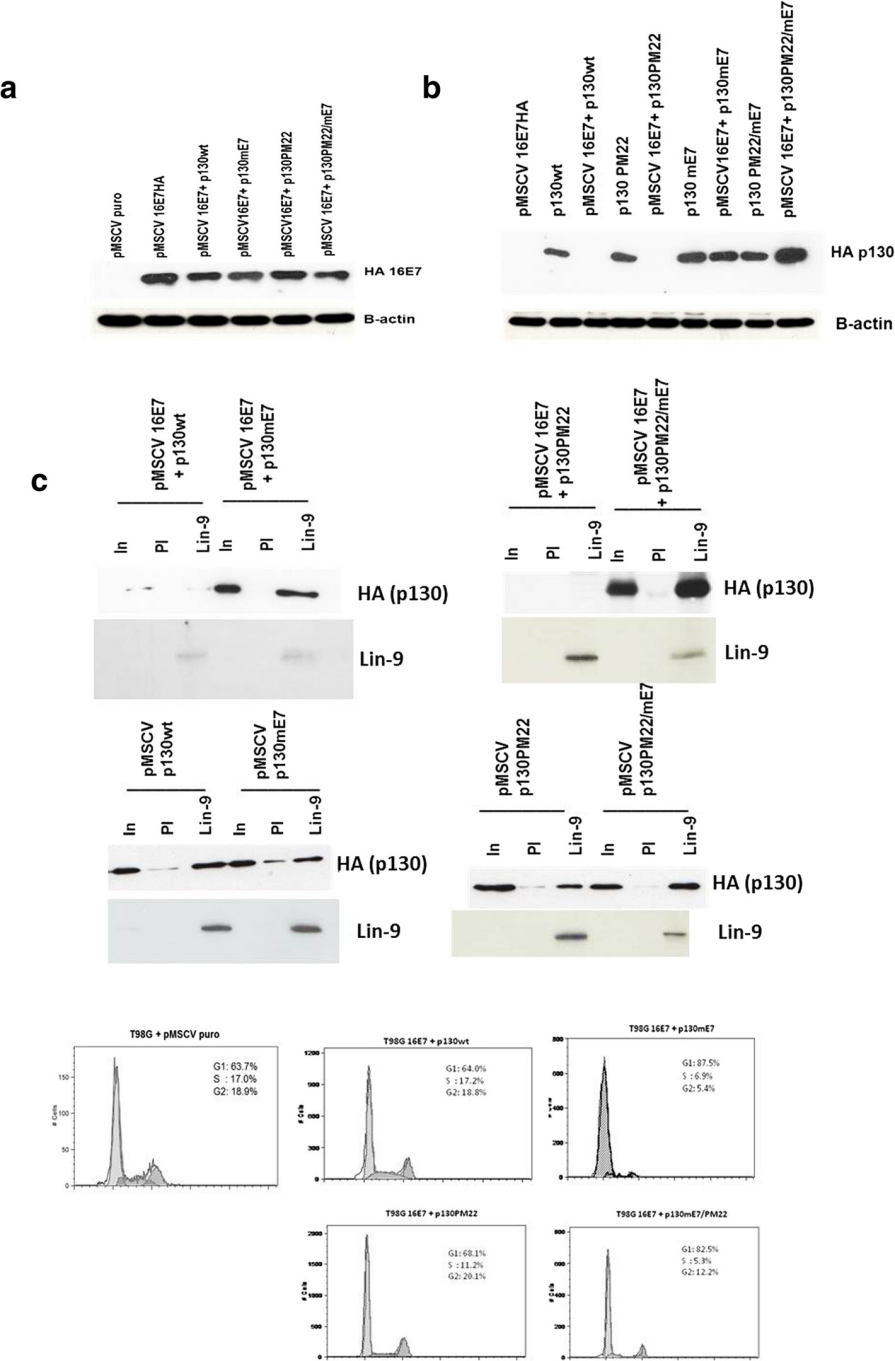
The LXCXE motif of HPV16 E7 binds to p130. **a** 20 μg of each pMSCVpuro-16E7-HA was calcium phosphate co-transfected with each type of p130 mutants for transient expression in T98G cells. In addition, the pMSCVpuro vector was transfected in T98G cells as a control. Transfected cells were puromycin selected and nuclear lysates were harvested 48 hours post transfection. Nuclear lysates were immunoprecipitated with HA (Roche) antibody, separated on a 15 % SDS-PAGE gel and western blotted onto a PVDF membrane. 16E7 proteins were detected using HA (Roche) antibody. The image is representative of three independent experiments. **b** Nuclear lysates from T98G cells transfected with pMSCV puro constructed with 16E7HA or p130 wt, p130 mE7, p130PM2, p130mE7/PM22 and a combination of 16E7 with each of the p130s. Transfected cells were puromycin selected and nuclear lysates were harvested 48 hours post transfection. Nuclear lysates were separated on a 10 % SDS-PAGE gel and western blotted onto a nitrocellulose membrane. p130 proteins were detected using HA (Roche) antibody. The image is representative of three independent experiments. **c** Detection of ectopically expressed p130 in immunoprecipitates of T98G cells transfected with pMSCV puro constructed with p130 wt, p130mE7, p130PM22, p130mE7/PM22 and a combination of 16E7 with each of the p130 constructs. Pre-immune serum (PI) and Lin-9 antibodies were used for immunoprecipitation and HA p130 was detected on western blots. The co-immunoprecipitation control, Lin-9 was detected using Lin-9 (Abcam) antibody. The image is representative of three independent experiments. (d) Flow cytometry of propidium iodide-stained T98G cells transfected with pMSCV puro constructed with p130wt, p130mE7, p130PM22, p130mE7/PM22 together with 16E7HA. The T98G cells transfected with pMSCV puro only was used as a control. The estimated percentages of cells in G1, S, and G2/M phase are shown

To confirm that HPV16 E7 targets p130 through its LXCXE motif, T98G (human glioblastoma) cells were transfected with pMSCVpuro vectors encoding HA-tagged p130wt, p130mE7, p130PM22 and p130PM22/mE7 together with 16E7. First, it was established that the p130 proteins were expressed equally and that expression of p130 had no effect upon 16E7 expression. The resultant western blot (Fig. 3a) showed expression of 16E7 with only minor variation. The p130wt and mutant proteins were also consistently expressed in T98G cells (Fig. 3b).

We then investigated what effect co-expression of 16E7 would have on the co-transfected p130 proteins. Whereas, co-expression with 16E7 had no significant impact on detection of p130mE7 or p130PM22/mE7 (Fig. 3b), it resulted in the virtual elimination of p130wt and p130PM22 expression. The loss of p130wt and p130PM22 expression in this experiment suggests that binding to 16E7 through the LXCXE motif resulted in proteasomal degradation, whereby the p130 proteins are degraded by the ubiquitin-proteasome pathway [19]. HPV16 E7 and p130 both interact with and are ubiquitylated by SCF^Skp2^ complex [20]. As anticipated, formation of the DREAM complex with p130wt and p130PM22 in T98G-transfected cells was significantly disrupted by 16E7, whereas this complex was abundantly expressed with p130mE7 and p130PM22/mE7 (Fig. 3c). Consistent with these results, analysis of T98G cell cycle status showed a profound G0/G1 arrest when p130mE7 and p130mE7/PM22 were co-transfected with 16E7, with 87.5 % and 82.5 % of cells in G0/G1, respectively, whereas 16E7 co-transfection abolished the ability of p130wt and p130PM22 to cause arrest (64 % and 68.1 % of cells in G0/G1) (Fig. 3d).

Studies in our laboratory have shown that the cutaneous HPV48 E7 protein was competent to bind to the CDK inhibitor (Cdki), p21^Cip1^*in vitro* (Additional file 2: Figure S2b). It is known that p21 together with ^p27Kip1^ are involved in E2F/pocket protein-cyclin E/A-CDK2 pathways in G1 and S phase. By binding to p21, HPV 48E7 will abrogate its inhibition of CDK2 activity, therefore potentially resulting in p130 hyperphosphorylation, and a block in its association with E2F4 and DREAM. However, the *in vitro* binding assay of 48E7 to p130wt and p130mE7 was not performed since 48E7 lacks a functional LXCXE domain. Previously, this observation has been carried out by Caldeira et al. between 48E7 and pRB or p107.

**Fig. 4:**
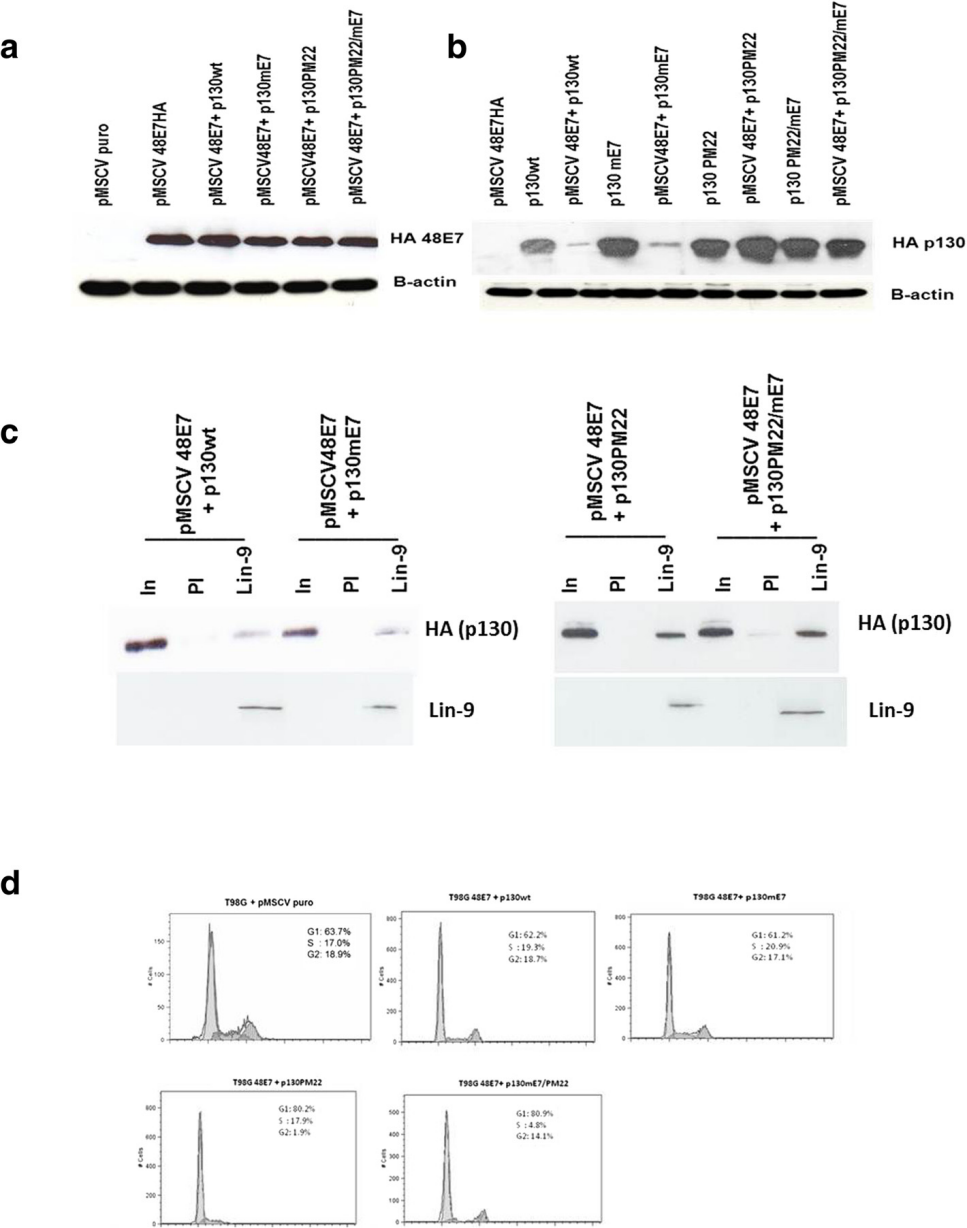
Co-expression of HPV 48E7 with p130wt and p130mE7 disrupt the p130-DREAM complexes. **a** 20 μg of each pMSCVpuro-48E7-HA was calcium phosphate co-transfected with each type of p130mutants for transient expression in T98G cells. In addition, the pMSCVpuro vector was transfected in T98G cells as a control.Transfected cells were puromycin selected and nuclear lysates were harvested 48 hours post transfection. Nuclear lysates were immunoprecipitated with HA (Roche) antibody, separated on a 15 % SDS-PAGE gel and western blotted onto a PVDF membrane. 48E7 proteins were detected using HA (Roche) antibody. The image is representative of four independent experiments. **b** Nuclear lysates from T98G cells transfected with pMSCV puro constructed with 48E7HA or p130 wt, p130 mE7, p130PM2, p130mE7/PM22 and a combination of 48E7 with each of the p130s. Transfected cells were puromycin selected and nuclear lysates were harvested 48 hours post transfection. Nuclear lysates were separated on a 10 % SDS-PAGE gel and western blotted onto a nitrocellulose membrane. HA p130 antibody was detected on western blots. The image is representative of three independent experiments. **c** Detection of ectopically expressed p130 in immunoprecipitates of T98G cells transfected with pMSCV puro constructed with p130 wt, p130mE7, p130PM22, p130mE7/PM22 and a combination of 48E7 with each of the p130 constructs. Pre-immune serum (PI) and Lin-9 antibodies were used for immunoprecipitation and HA p130 was detected on western blots. The image is representative of three independent experiments. **d** Flow cytometry of propidium iodide-stained T98G cells transfected with pMSCV puro constructed with p130wt, p130mE7, p130PM22, p130mE7/PM22 together with 48E7HA. The T98G cells transfected with pMSCV puro only was used as a control. The estimated percentages of cells in G1, S, and G2/M phase are shown

To explore whether our previous finding [21] that 48E7 can disrupt the p130-DREAM complex reflects direct binding to p130 or alternatively an effect on CDK activity, T98G cells were transfected with 48E7 and the HA-tagged wt and mutant p130 proteins used in the previous experiments. We first established that HPV48 E7 was expressed consistently in each transfection (Fig. 4a). Next we demonstrated that both p130wt and p130mE7 were degraded when 48E7 was co-expressed, whereas p130PM22 and p130PM22/mE7 were unaffected by 48E7 (Fig. 4b). These findings indicated that 48E7 suppresses p130 expression through CDK phosphorylation. This activity most probably reflects binding of 48E7 to p21and degradation of the resultant hyperphosphorylated p130.

We also found that the p130-DREAM complex was significantly reduced when p130wt and p130mE7 were co-expressed with 48E7, compared to formation of this complex with p130PM22 or p130PM22/mE7 (Fig. 4c). To determine what impact this would have on the ability of the various p130 proteins to arrest the T98G cell cycle, the transfected cells were harvested and subjected to propidium iodide staining. Flow cytometry analysis showed that cells arrested at G0/G1 when either p130PM22 or p130PM22/mE7 were co-expressed with 48E7 (80.2 % and 89.9 % cells in G0/G1, respectively), whereas cells expressing p130wt or p130mE7 were able to escape arrest in the presence of 48E7 (Fig. 4d).

Taken together, our results show that both 16E7 and 48E7 proteins lead to decrease steady state levels of p130, diminish p130-DREAM complexes and promote the cell to enter S-phase, but that this occurs by completely different mechanisms. More specifically, the results suggest that HPV16 E7 targets p130 predominantly through direct interactions via the LXCXE motif whereas HPV48 E7 disrupts p130-DREAM via CDK2 phosphorylation of p130. It is significant that whereas different HPV E7 types have a wide range of affinity towards pocket protein binding, based on their LXCXE motif [21], HPV48 E7 (which lacks a functional LXCXE motif) demonstrates similar effects on p130-DREAM formation and cell cycle progression compared to HPV16 E7. Pang et al. (2014) reported that the LXCXE motif of HPV16 E7 could promote mitotic gene expression by a pocket protein-independent mechanism; this was associated with 16E7 binding to B-Myb, FoxM1 and Lin9 which associate in a transcription activating form of the DREAM complex (dubbed B-Myb-MuvB) present during S-G2. It is to be expected that HPV48 E7 would be unable to activate B-Myb-MuvB, and would therefore lack the ability to induce mitotic gene expression as does HPV16 E7.

## Conclusions

Our studies indicate that HPV E7 proteins which differ markedly in their ability to interact directly with pocket proteins can both cause p130 degradation and lead to p130-DREAM disruption, resulting in promotion of cell proliferation. Whereas HPV16 E7 directly interacts with p130 through its LXCXE motif, HPV48 E7 (which lacks a functional LXCXE motif) alternatively might suppresses p130 through CDK phosphorylation. That both mechanisms exist, demonstrates that targeting p130-DREAM is imperative for HPV types to induce cell cycle progression.

## Additional files

Additional file 1: Figure S1. Expression of various p130 mutants in C33a and CaSki cell lines. pMSCV puro (Clontech, Mountain View, CA) constructed with p130 wt and p130 mutants (p130mE7, p130PM22 and p130 mE7/PM22) were ‘FuGENE 6’ transfected in C33a and CaSki cells. The T98G cell lines (HPV 16 E7 negative cell line) were used as a control cells. Transfected cells were puromycin selected and nuclear lysates were harvested 48 hours post transfection. Nuclear lysates were separated on a 10 % SDS-PAGE gel and western blotted onto a nitrocellulose membrane. (a) Endogenous p130 were detected by p130 (Santa Cruz) and β-actin (Sigma Aldrich) was used as a loading control. (b) Ectopically expressed p130 were detected by p130 (Santa Cruz) and HA (Roche) antibodies, respectively. β-actin (Sigma Aldrich) was used as a loading control. The image is representative of three independent experiments. (JPG 51 kb) Additional file 2: Figure S2. The p130mE7 mutant is unable to bind 16E7 and HPV48 E7 binds to p21. (A) Nuclear extracts were prepared from T98G cells transfected with the pMSCVpuro vector encoding HA-tagged p130wt or p130mE7 and from cells transfected with the empty vector (con). Nuclear extracts (150 μg) were incubated with 20 μg 16E7 protein bound to glutathione-Sepaharose beads and the selected proteins were eluted and run on a western blot alongside inputs comprising 15 μg of each nuclear extract. The p130wt and p130mE7 were detected using an HA antibody probe. In addition to the p130 proteins, a non-specific (n.s.) band was seen with the input samples. (B) A GST binding assay was carried out using GST-tagged-E7- HA proteins and IVT [^35^S]-labelled p21. 25 μg of each GST16-E7-HA and GST48-E7-HA proteins were resolved by SDS-PAGE alongside inputs comprising 15 μg of nuclear extract and were detected using an HA antibody probe. Any bound p21 was visualised by autoradiography. The image is representative of three independent experiments. (JPG 35 kb)

## Abbreviations

HPV: Human papillomavirus
RBL2: Retinoblastoma like protein 2
CDK2: Cyclin dependent kinase 2
HDACs: Histone deacytelases
