# Supplementary figures and images for: HPV 16E7 and 48E7 proteins use different mechanisms to target p130 to overcome cell cycle block
Source: Virol J. 2016 Jan 4;13:2. doi: 10.1186/s12985-015-0460-8 (PMC4700559; doi:10.1186/s12985-015-0460-8)

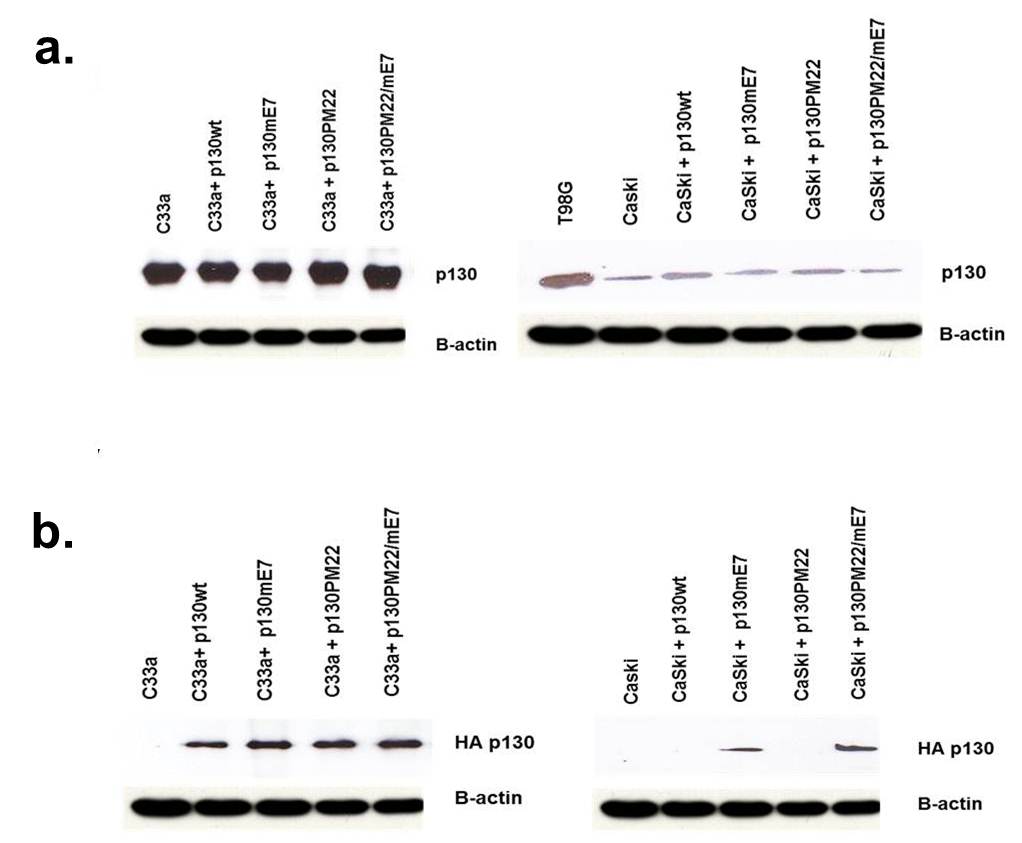

Supplement: Additional file 1: Figure S1. — Expression of various p130 mutants in C33a and CaSki cell lines. pMSCV puro (Clontech, Mountain View, CA) constructed with p130 wt and p130 mutants (p130mE7, p130PM22 and p130 mE7/PM22) were ‘FuGENE 6’ transfected in C33a and CaSki cells. The T98G cell lines (HPV 16 E7 negative cell line) were used as a control cells. Transfected cells were puromycin selected and nuclear lysates were harvested 48 hours post transfection. Nuclear lysates were separated on a 10 % SDS-PAGE gel and western blotted onto a nitrocellulose membrane. (a) Endogenous p130 were detected by p130 (Santa Cruz) and β-actin (Sigma Aldrich) was used as a loading control. (b) Ectopically expressed p130 were detected by p130 (Santa Cruz) and HA (Roche) antibodies, respectively. β-actin (Sigma Aldrich) was used as a loading control. The image is representative of three independent experiments. (JPG 51 kb) [file 12985_2015_460_MOESM1_ESM.jpg]

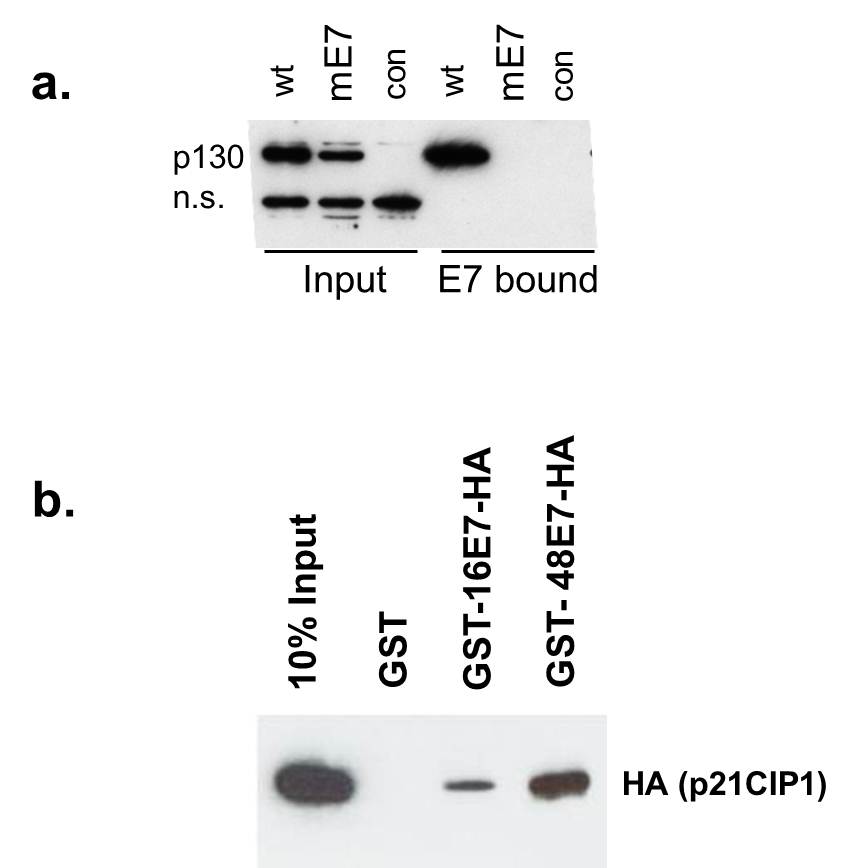

Supplement: Additional file 2: Figure S2. — The p130mE7 mutant is unable to bind 16E7 and HPV48 E7 binds to p21. (A) Nuclear extracts were prepared from T98G cells transfected with the pMSCVpuro vector encoding HA-tagged p130wt or p130mE7 and from cells transfected with the empty vector (con). Nuclear extracts (150 μg) were incubated with 20 μg 16E7 protein bound to glutathione-Sepaharose beads and the selected proteins were eluted and run on a western blot alongside inputs comprising 15 μg of each nuclear extract. The p130wt and p130mE7 were detected using an HA antibody probe. In addition to the p130 proteins, a non-specific (n.s.) band was seen with the input samples. (B) A GST binding assay was carried out using GST-tagged-E7- HA proteins and IVT [35S]-labelled p21. 25 μg of each GST16-E7-HA and GST48-E7-HA proteins were resolved by SDS-PAGE alongside inputs comprising 15 μg of nuclear extract and were detected using an HA antibody probe. Any bound p21 was visualised by autoradiography. The image is representative of three independent experiments. (JPG 35 kb) [file 12985_2015_460_MOESM2_ESM.jpg]
